# Exploring the prevalence and risk factors of peripheral artery disease in patients with type 2 diabetes in sub-Saharan Africa: a systematic review and meta-analysis

**DOI:** 10.3389/fcdhc.2025.1563984

**Published:** 2025-07-21

**Authors:** Kirubel Eshetu Haile, Atitegeb Alebachew Amsalu, Gizachew Ambaw Kassie, Yordanos Sisay Asgedom, Gedion Asnake Azeze, Amanuel Yosef Gebrekidan

**Affiliations:** ^1^ School of Nursing, College of Health Sciences and Medicine, Wolaita Sodo University, Wolaita Sodo, Ethiopia; ^2^ School of Public Health, College of Health Sciences and Medicine, Wolaita Sodo University, Wolaita Sodo, Ethiopia; ^3^ School of Midwifery, College of Health Sciences and Medicine, Hawassa University, Hawassa, Ethiopia

**Keywords:** peripheral artery disease, lower extremity, diabetes mellitus, sub-Saharan Africa, type 2 diabetes mellitus

## Abstract

**Background:**

Type 2 diabetes and lower-extremity peripheral artery disease (PAD) are growing global health problems associated with considerable cardiovascular and limb-related morbidity and mortality, poor quality of life, and high healthcare resource use and costs. Diabetes is a well-known risk factor for PAD, which further increases the risk of long-term complications. The primary aim of this systematic review was to ascertain the aggregated prevalence of PAD among individuals diagnosed with type 2 diabetes mellitus (T2DM) residing in sub-Saharan Africa.

**Objective:**

The aim of this study was to determine the pooled prevalence and associated factors of PAD among patients with T2DM in sub-Saharan Africa.

**Methods:**

A systematic review and meta-analysis was performed in alignment with the guidelines established by the Preferred Reporting Items for Systematic Reviews and Meta-Analyses. To identify papers published in English up to 8 November 2024, the electronic databases of Medline, Web of Science, Science Direct, Excerpta Medica Database, Cochrane Library, African Journals Online, and Google Scholar were searched. A random-effects model was employed to estimate the pooled prevalence and associated factors of PAD.

**Results:**

This study revealed that the pooled prevalence of PAD among patients with T2DM was 35.7% [95% confidence interval (CI) 28.7, 42.7], reflecting the significant impact of DM on vascular health with statistically significant heterogeneity observed between studies (*I* ^2^ = 94.9%, *p* < 0.001). Age, elevated low-density lipoprotein, elevated body mass index (BMI), and diabetes illness duration exceeding 10 years were the significant predictors.

**Conclusion:**

The aggregate burden of PAD in individuals with T2DM within the sub-Saharan African region is estimated at 35.7%, suggesting that a considerable segment of the sub-Saharan population has been impacted. Epidemiological studies utilizing precise assessment tools can enhance the early detection and prevention of PAD in T2DM and improve the certainty of findings.

**Clinical implication:**

There is a need for integrated care approaches that prioritize the screening and management of PAD in individuals with T2DM. Given the high prevalence and associated complications, healthcare providers should implement routine PAD assessments in diabetes care protocols. Future research should focus on longitudinal studies that explore the causal relationships between risk factors and the development of PAD in patients with T2DM.

**Systematic Review Registration:**

https://www.crd.york.ac.uk/prospero, identifier CRD42024611838.

## Introduction

Diabetes mellitus (DM) is an escalating public health concern associated with significant rates of cardiovascular risk as well as limb-related morbidity and mortality. In 2021, an estimated 4.5% of the adult population aged 20–79 years in sub-Saharan Africa was affected by diabetes, which equates to approximately 24 million individuals. Projection indicated that by the year 2045, this number is predicted to increase by 129% to a total of 55 million, marking the highest increase among all the regions delineated by the International Diabetes Federation (IDF). In 2017, the prevalence of diabetes within sub-Saharan Africa was approximately 6%, with an estimated 15.5 million adults (ranging from 9.8 to 27.8 million) diagnosed with diabetes, resulting in the healthcare expenditure estimated at USD 3.3 billion (1, 2). Notably, over 54% of people with diabetes in sub-Saharan Africa remain undiagnosed, representing the highest rate on a global scale. In 2017, the countries with the highest number of people with diabetes (age range from 18 to 99 years) were Ethiopia, South Africa, the Democratic Republic of Congo, Nigeria, and Tanzania. This prevalence is expected to increase by 162.5% by the year 2045, with an estimated 40.7 million adults affected by type 2 diabetes and healthcare expenditures potentially reaching USD 6 billion (3–5).

The global prevalence of type 2 diabetes mellitus (T2DM) is witnessing a rapid and significant growth. The recent statistics reveal that as of the year 2021, approximately 529 million individuals across the globe have been diagnosed with T2DM, with a remarkable 96% of these individuals with diabetes being elderly adults (6). The extensive prevalence of T2DM is accompanied by numerous micro- and macrovascular complications, one of which is peripheral artery disease (PAD), with an estimated prevalence rate of 14% (7). This condition is characterized by atherosclerotic narrowing of the artery in the lower extremities, leading to impaired distal perfusion, primarily induced by atherosclerosis, which culminates in diminished functional capacity, and substantial morbidity and mortality (8, 9).

PAD ranks as the third most prevalent atherosclerotic condition following coronary artery disease and stroke. In 2015, approximately 236 million people were affected by PAD, marking a 17.1% increase from the prevalence rate reported in 2010 (9, 10). According to data from the American Diabetes Association (ADA), it has been observed that African American and Hispanic populations exhibit the highest rates of coexisting PAD and DM (11).

Patients with PAD and their treating healthcare professionals do not recognize functional impairment as a clinical future of vascular disease, 30%–50% of patients have atypical exertional leg symptoms, and only 20% suffer from intermittent claudication (12–14). It is also the most commonly underdiagnosed disease, especially among patients with T2DM, because most patients with PAD are asymptomatic and do not complain of intermittent claudication due to decreased pain perception secondary to peripheral neuropathy, which could lead to adverse cardiovascular outcomes, limb-related morbidity and mortality, poor quality of life, amputations, acute limb ischemia, infections, acute occlusions, ulcers, high healthcare expenditures, increased length of hospital stay, and increased admission and readmission rates (5, 14).

The prevalence of PAD among patients with T2DM is significant, and various predictors enhance its development and progression. People with diabetes are three to four times more likely to develop PAD than those without diabetes. Furthermore, in patients with T2DM, the presence of PAD significantly exacerbates the risk of lower limb complications, such as leg amputation, revascularization procedures, cardiovascular complications, and elevated mortality rates (9, 10). Research conducted across the globe has shown differing prevalence rates, with a higher rate reported in the African region due to varying lifestyle factors and healthcare access. According to a systematic literature review, patients with T2DM have a 12.5%–22% prevalence of PAD, which is associated with increased morbidity and mortality (5). In studies conducted in Korea (15), and India (16), the prevalence of PAD among patients with DM was 28.7% and 36%, respectively.

A multi-country study on the prevalence and clinical features of PAD in Asian patients with T2DM found a prevalence of 17.7% (17). However, studies in Karnataka, India, and America reported a lower prevalence of PAD, ranging between 7.5% and 8.5% (17, 18). Conversely, some studies in India and Pakistan found prevalence rates between 30% and 40% (19). In a study conducted in sub-Saharan Africa, approximately 38.5% of patients with T2DM were found to have PAD (20). The prevalence of PAD among individuals with T2DM in sub-Saharan Africa demonstrates notable gender differences. Research indicates that men generally exhibit a higher prevalence of PAD than women. Studies report prevalence rates of PAD in men with T2DM ranging from 35% to 50% in various sub-Saharan African countries, and among women, the prevalence rate tends to be lower, typically approximately 20% to 30%, as high as 35% in specific populations (21).

However, previously available evidence has focused on the prevalence of PAD in the overall population; comprehensive data on the pooled prevalence of PAD and associated factors among patients with T2DM in sub-Saharan African countries are lacking. Furthermore, methodological discrepancies in research design, diagnostic criteria, and biomarker utilization impede comparative evaluations, resulting in a scarcity of high-quality data. Therefore, our intent was to conduct a systematic review and meta-analysis of all available studies to determine the current pooled prevalence of PAD and associated factors among patients with T2DM in sub-Saharan African countries. Overall, comprehending the pooled prevalence and management of PAD is critical for healthcare systems to formulate effective and efficient preventive strategies aimed at reducing the risks linked to PAD in individuals diagnosed with T2DM.

## Methods

### Study design and reporting

A systematic review and meta-analysis of observational studies was conducted on the burden of PAD and its associated factors among patients with T2DM in sub-Saharan Africa. All studies on PAD and its associated factors among patients with DM in sub-Saharan Africa published up to 8 November 2024 were retrieved via the Preferred Reporting Items for Systematic Reviews and Meta-Analyses (PRISMA) guidelines (22).

### Protocol and registration

The review was registered in PROSPERO (International Prospective Register of Systematic Reviews), the University of York Center for Reviews and Dissemination with registration number CRD42024611838.

### Search strategy

We conducted a systematic and comprehensive search of the electronic databases of ScienceDirect, Cochrane Library, Medline, Center for Evidence-based Medicine, African Journals Online (AJOL), Excerpta Medical Database, Web of Science, Scopus, and Google Scholar to identify all relevant observational studies on the prevalence of PAD and associated factors among patients with T2DM in a sub-Saharan African region up to 8 November 2024. The articles were downloaded, arranged, and referenced via the EndNote referencing manager version 20. To find more potentially relevant studies, a manual search was conducted using the reference lists of the retrieved articles. Research was restricted to works published in the English language. The search was carried out using the following keywords: “prevalence” (“Peripheral artery disease,” “PAD,” OR “lower extremity artery disease” OR “arterial stiffness”) and (people or patients OR individuals OR male OR female OR adults) and Diabetes Mellitus OR diabetic OR type 2 diabetes Mellitus) OR (associated Factors OR determinant factors OR risk factors OR outcome) AND (epidemiology OR prevalence OR incidence) AND (Sub-Saharan Africa OR east Africa OR west Africa OR South Africa OR central Africa). The search terms were used separately and in Combination with “OR” or “AND” (**Supplementary File 1**).

We considered studies that examined the prevalence of PAD and associated factors among patients with T2DM in sub-Saharan Africa to be relevant. Our imposed search limits restricted studies that were observational in nature, those published in English, and those that involved human participants. The PubMed search engine with MeSH (Medical Subject Headings) and Boolean databases were searched.

### Eligibility criteria

#### Inclusion criteria

Studies were included if they met the following requirements: (i) study period: studies conducted or published until 8 November 2024; (ii) study type: observational studies (cross-sectional); (iii) population: research conducted on T2DM; (iv) outcome: prevalence (proportion); (v) place of study: research conducted in sub-Saharan Africa; and (vi) studies published in the English language.

PICO: population: people with T2DM; intervention: not applicable; comparison: not applicable; outcome: PAD.

#### Exclusion criteria

Studies were excluded based on the following criteria: (1) studies with incomplete data; (2) inaccessibility of the full-text article; and (3) studies consisting of review articles, case series, case reports, and letters to the editors were not included.

### Definition of PAD

All extracted studies included a definition of PAD. Twelve studies relied on ankle–brachial index (ABI) values ≤ 0.9 (either alone or in combination with other parameters) (20, 23–33), while one study utilized color Doppler ultrasonography as diagnostic criteria (34).

### Study selection and extraction

**Figure 1:**
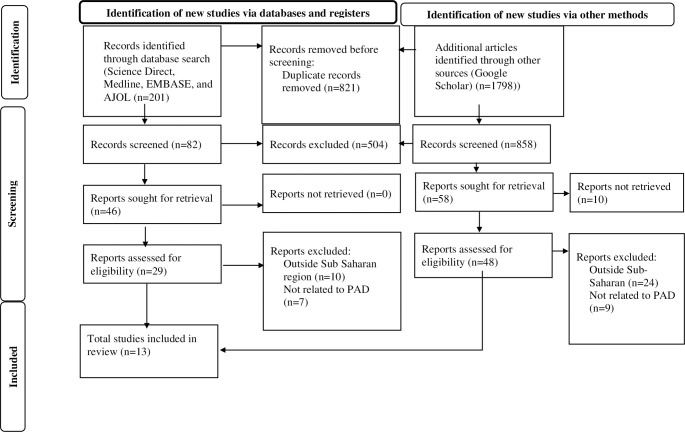
PRISMA flow diagram of the selection process of studies on peripheral Artery disease among T 2DM patients in Sub-Saharan Africa.

The retrieved studies were imported into EndNote (Version 20, for Windows, Thomson Reuters, Philadelphia, PA, USA), and Endnote was used to eliminate 821 duplicate studies. Three independent reviewers (KEH, AAA, and GAK) screened all papers for eligibility requirements: first, the abstracts and titles were screened, and then, the full texts were screened. Three investigators (YSA, AYG, and GAA) independently used a consistent data extraction format created in Microsoft Excel to extract data. Prior to the extraction process, the three independent researchers were blinded to the study data. Name of the first author, year of publication, country, subcontinent, sample size, and response rate (**Figure 1**, **Table 1**).

**Table 1:** Characteristics of the 13 studies included in the systematic review and meta-analysis of PAD among patients with T2DM in Sub-Saharan Africa.

Study No	author	year	Country	Sub-Continent	Study design	Population	prevalence	Sample Size	RR(%)
1	Akalu.Y et.al	2020	Ethiopia	East Africa	cross-sectional	T2DM	30.7	280	100
2	swali.A Et al	2017	Tanzania	East Africa	cross-sectional	T2DM	30.7	367	100
3	Osman.A et al	2023	Sudan	East Africa	cross-sectional	T2DM	22	100	100
4	Agboghoroma et.al	2020	Nigeria	West Africa	cross-sectional	T2DM	38.5	200	100
5	Immanuel.A et	2016	Ghana	West Africa	cross-sectional	T2DM	35.5	200	100
6	PD. Y et al	2018	Nigeria	West Africa	cross-sectional	T2DM	29	200	100
7	ACA.C et al	2019	Nigeria	West Africa	cross-sectional	T2DM	31.1	405	100
8	Umuerri. EM et.al	2013	Nigeria	West Africa	cross-sectional	T2DM	35.6	388	100
9	Christian, A et al	2024	Gabon	Central Africa	cross-sectional	T2DM	34.24	219	100
10	Awadalla H et al	2017	Sudan	East Africa	cross-sectional	T2DM	68.2	424	100
11	Oyewole. O et al	2024	Nigeria	West Africa	cross-sectional	T2DM	33.8	210	100
12	Ikem R et al	2022	Nigeria	West Africa	cross-sectional	T2DM	42.5	400	100
13	Rheeder, P et al	2004	South Africa	South Africa	cross-sectional	T2DM	30.4	112	85

### Quality assessment

Following the full text review, the Newcastle–Ottawa quality assessment scale (which was modified for cross-sectional studies) was used by three authors (AYG, GAK, and YSA) to evaluate the article’s quality (35). Disagreements were settled by consensus and discussion. We employed the following elements as criteria for an appraisal: (1) the degree of representativeness of the sample (maximum score = 1), (2) the magnitude of the sample size (maximum score = 1), (3) the incidence of non-respondents (maximum score = 1), (4) the method of ascertainment of the exposure (risk factor) (maximum score = 2), (5) the number of subjects in different outcome groups being comparable, on the basis of the study design (maximum score = 2), (6) the assessment of outcome (maximum score = 2), and (7) the statistical test (maximum score = 1). Articles with a score of ≥5 on the quality evaluation checklist criteria were considered low-risk studies, and these studies were included in the systematic review and meta-analysis. No study was excluded after a quality rating was obtained (**Table 2**).

**Table 2:** Quality rating for studies included in the systematic review and meta-analysis of PAD among patients with T2DM in sub-Saharan Africa region.

Study	Selection Criteria				Comparability	Outcome		
	Representativeness of the sample *	Sample size *	Non respondents *	Ascertainment of the exposure (maximum score=2) **	The subjects in different outcome groups are comparable, based on the study design or analysis. Confounding factors are controlled (maximum score=2) **	Assessment of the outcome (maximum score=2) **	Statistical test *	Total (10)
Akalu.Y et.al	1	1	1	2	1	1	1	8
swali.A Et al	1	1	1	2	1	1	1	8
Osman.A et al	1	0	1	2	1	1	1	7
Agboghoroma et.al	1	1	1	2	1	1	1	8
Immanuel.A et	1	1	1	1	1	1	1	7
PD. Y et al	1	1	1	1	1	1	1	7
ACA.C et al	1	1	1	2	1	1	1	8
Umuerri. EM et.al	1	1	1	1	1	1	1	7
Christian, A et al	1	1	1	1	1	1	1	8
Awadalla, H et al	1	1	1	1	1	1	1	7
Oyewole. O et al	1	1	1	1	1	1	1	7
Ikem R et al	1	1	1	2	1	1	1	8
Rheeder, P et al	1	1	1	1	1	1	1	7

score=1, and * score=2)

### Statistical analysis

The data were analyzed via STATA 14.2 software (StataCorp, College Station, Texas, USA). We used the DerSimonian and Laird method for random-effects models to calculate the pooled prevalence of PAD among patients with type 2 DM in sub-Saharan African countries (36). The *I* ^2^ statistical test was conducted to check heterogeneity across studies. *I* ^2^ values of 0%, 25%, 50%, and 75% were assumed to denote the absence of heterogeneity, a low level of heterogeneity, a moderate degree of heterogeneity, and a high degree of heterogeneity, respectively. Because significant heterogeneity was detected between studies, a meta-analysis using a random-effects model was conducted to estimate pooled prevalence of PAD with 95% confidence intervals (CIs). A forest plot was used to present the results of the meta-analysis. The Egger’s test was employed to assess the existence of publication bias, and any possible publication bias was specified by visual examination of the funnel plot.

To pinpoint the key studies that had the most significant influence on between-study heterogeneity, a leave-one-out sensitivity analysis was also conducted. By omitting each study individually, an analysis was conducted to determine the impact of each study on the pooled estimated prevalence of patients with PAD and T2DM in sub-Saharan Africa. The input variables needed by the cells of the two-by-two tables for factors related to PAD are binary data, or “prevalence of peripheral artery disease,” i.e., the proportion of patients in each study’s exposed and non-exposed groups who have and do not have PAD. The odds ratio (OR), which was calculated on the basis of the binary results of the included main studies, was used to evaluate all factors associated with PAD. The pooled OR was calculated via random-effects meta-analysis, and a 95% CI was employed. The effect magnitude and 95% CIs are shown through forest plots.

## Results

A total of 2,820 scholarly articles were obtained, which were published until 8 November 2024, through the use of electronic databases. In total, 821 articles were excluded because of duplication. Of the remaining 1,999 articles, 1,912 were removed by title and abstract, whereas 87 were read in full and assessed for eligibility. On the other hand, no study that fulfilled the eligibility criteria was excluded, which failed to access the full text. Finally, 13 studies with a total of 3,505 participants who fulfilled the eligibility requirements were included in the meta-analysis (**Figure 1**, **Table 1**).

### Characteristics of the included studies

Among the 13 included studies, 7 were conducted in Western Africa (20, 23, 24, 26, 29, 30, 33), 4 were conducted in Eastern Africa (27, 28, 31, 34), 1 study was conducted in Central Africa (32), and the remaining study was conducted in South Africa (25). The highest prevalence (68.2) of PAD among individuals with T2DM in sub-Saharan Africa was reported by a study conducted in Sudan (27). The lowest prevalence (22) was reported in a study conducted in Sudan (31).

### Pooled burden of PAD among patients with T2DM in sub-Saharan Africa

**Figure 2:**
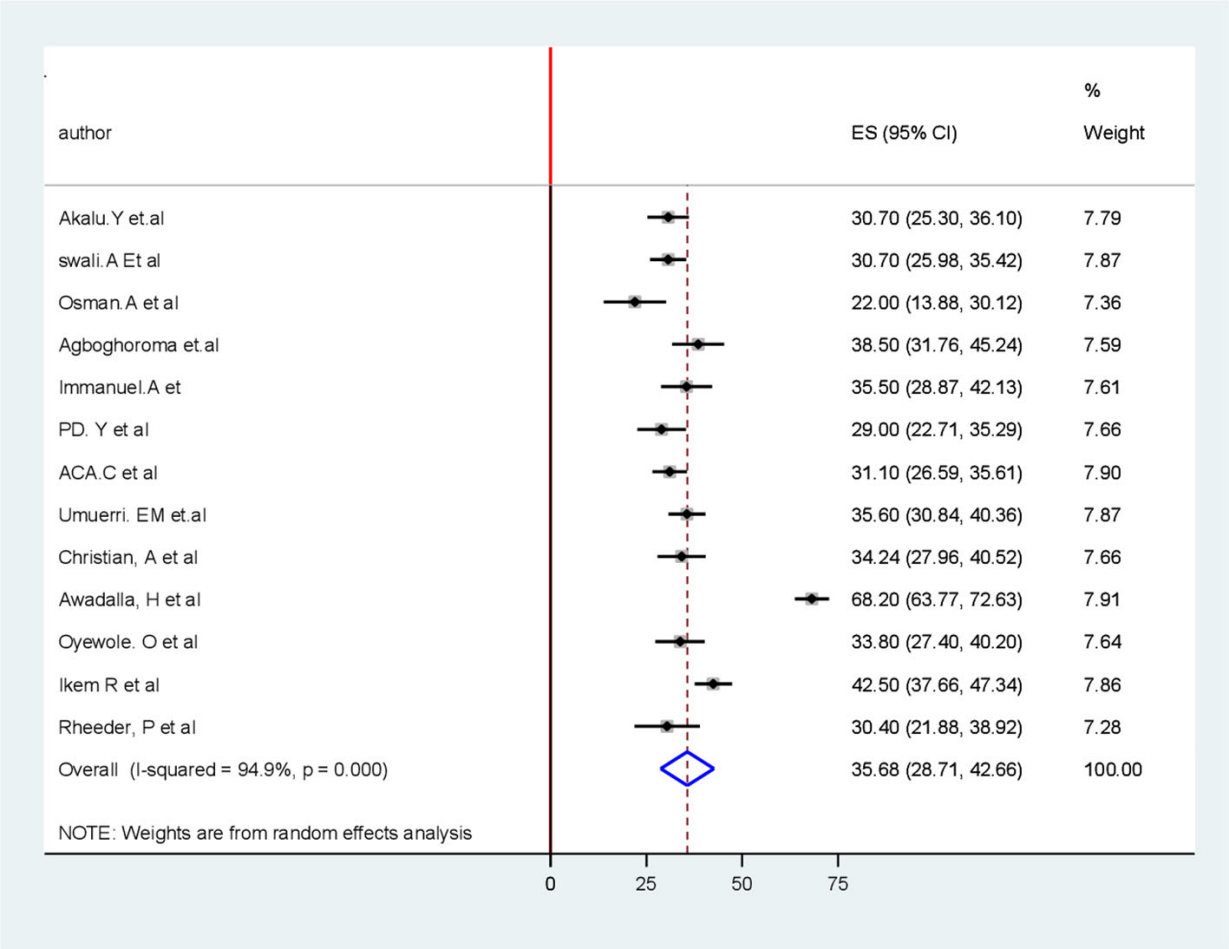
Forest plot for pooled burden of the systematic review and meta-analysis of peripheral Artery disease among patients with T2 DM in Sub-Saharan Africa.

The pooled prevalence of PAD among patients with T2DM in sub-Saharan African countries was 35.7% (95% CI 28.7, 42.7). The forest plot in **Figure 2** shows a statistically significant heterogeneity (*I* ^2^ = 94.9%; *p* < 0.001) (**Figure 2**). Therefore, a random-effects model was used to estimate the pooled prevalence of PAD among patients with T2DM in sub-Saharan African countries. Additionally, subgroup analysis was conducted to determine the potential source of heterogeneity among the studies because of the high degree of heterogeneity.

### Subgroup analysis

**Figure 3:**
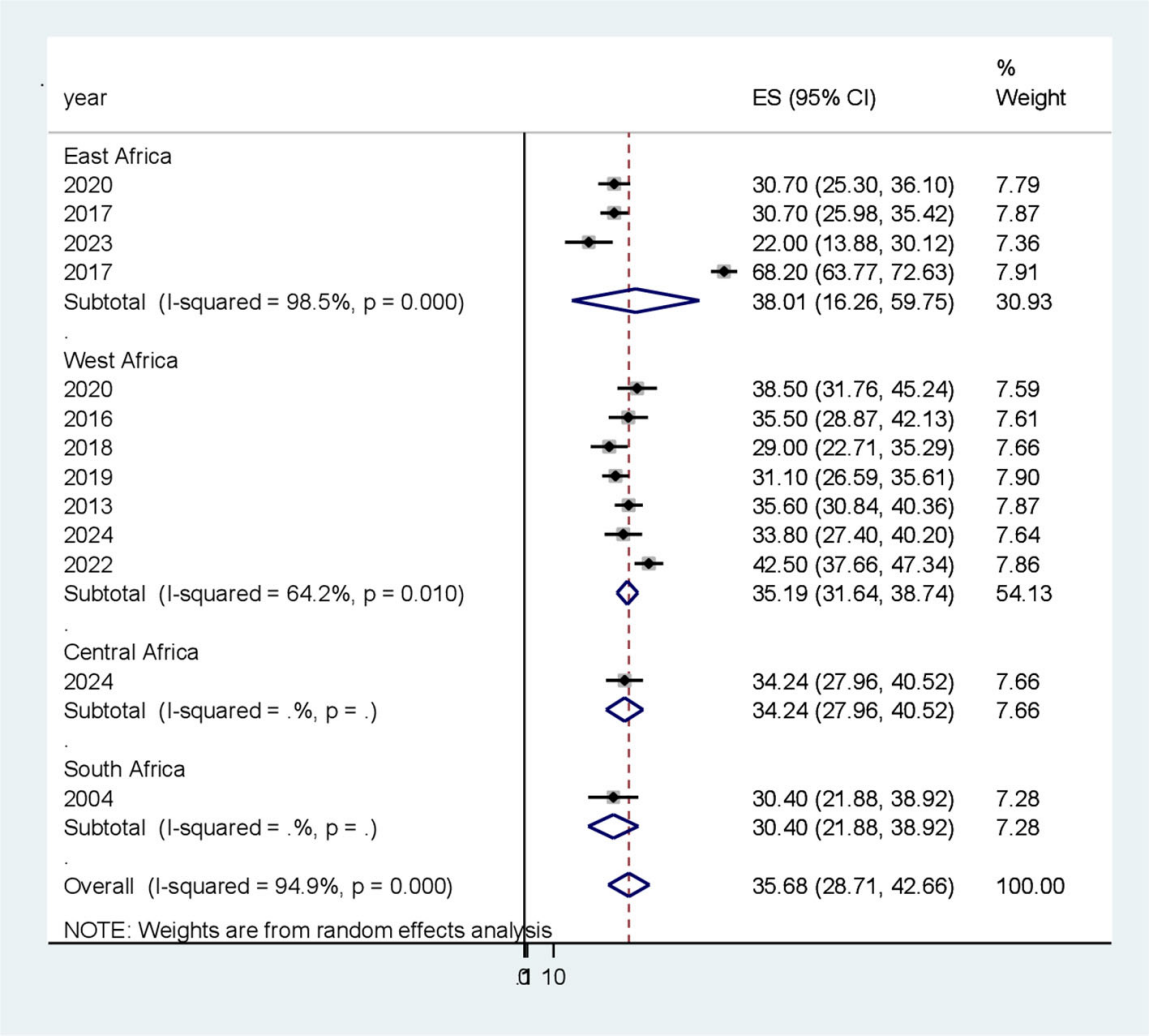
Subgroup analysis based on study area (sub-continent) for the systematic review andmeta-analysis of peripheral artery disease among patients with T2DM in sub-Saharan Africa.

Because of the significant heterogeneity among the studies, a subgroup analysis was conducted to identify potential sources of heterogeneity. Subgroup analysis was conducted depending on the study area (subcontinent) (**Figure 3**) and year of publication to identify possible sources of heterogeneity. With respect to the subgroup analysis by subcontinent, the highest pooled prevalence of PAD among patients with T2DM was reported in the Eastern African subcontinent at 38.00% (95% CI 16.26, 59.75), whereas the lowest was documented in the South African subcontinent at 30.4% (95% CI 21.9, 38.92) (**Table 3**).

**Table 3:** Subgroup analysis of the pooled prevalence of PAD among patients with T2 DM in sub-Saharan Africa.

Subgroups	Number of studies	Prevalence (95% CI)	Heterogeneity statistics	P-value	I2	Tau2
Subcontinent						
East Africa	4	38 (16.26 to 59.75)	195.86	<0.0001	98.5	483.5
West Africa	7	35.2 (31.6 to 38.7)	16.77	0.01	64.2	14.56
Central Africa	1	34.2 (27.96 to 40.5)	–	<0.0001	0.0%	0.0%
South Africa	1	30.4 (21.9 to 38.92)	–	<0.0001	0.0%	0.0%
**Overall**	13	35.7 (28.71 to 42.7)	237.12	<0.0001	94.9	155.1

### Publication bias

**Figure 4:**
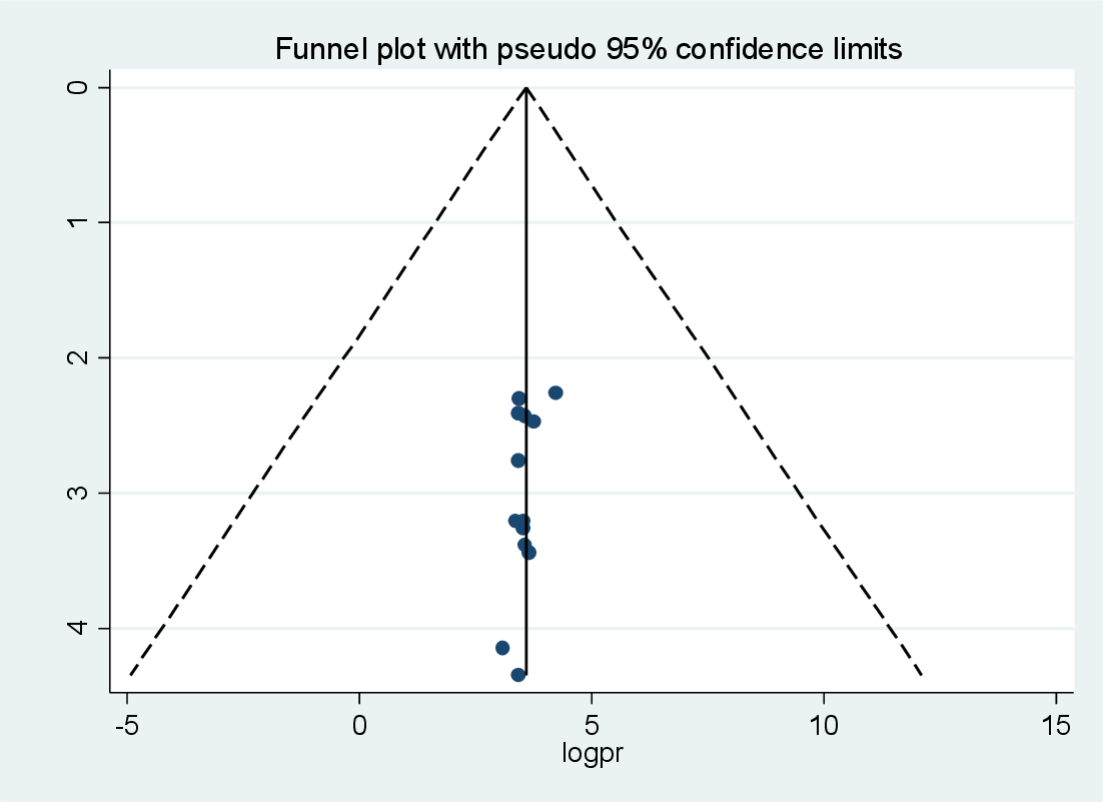
Funnel plot for publication bias for systematic review and meta-analysis of peripheralartery disease among patients with T2DM in sub-Saharan Africa.

The graphical presentation of the funnel plot indicates the presence of asymmetry (**Figure 4**). The results of the Egger test were statistically insignificant with coefficient = 4.28, 95% CI (3.39, 4.99) and with a *p*-value of 0.117 (**Table 4**). As a result, there are probably few missed studies.

**Table 4:** Tests for Funnel plot Asymmetry (Eggers test) of peripheral Artery disease among patients with type 2 diabetes mellitus in sub-Saharan Africa.

Standard effect	Coefficient	Standard error	T	P>t	95% CI
Slope	4.28	0.36	11.53	<0.0001	(3.39 to 4.99)
Bias	-0.217	0.13	-1.7	0.1	-(0.497 to 0.064)

### Sensitivity analysis

#### Leave-out-one sensitivity analysis

**Figure 5:**
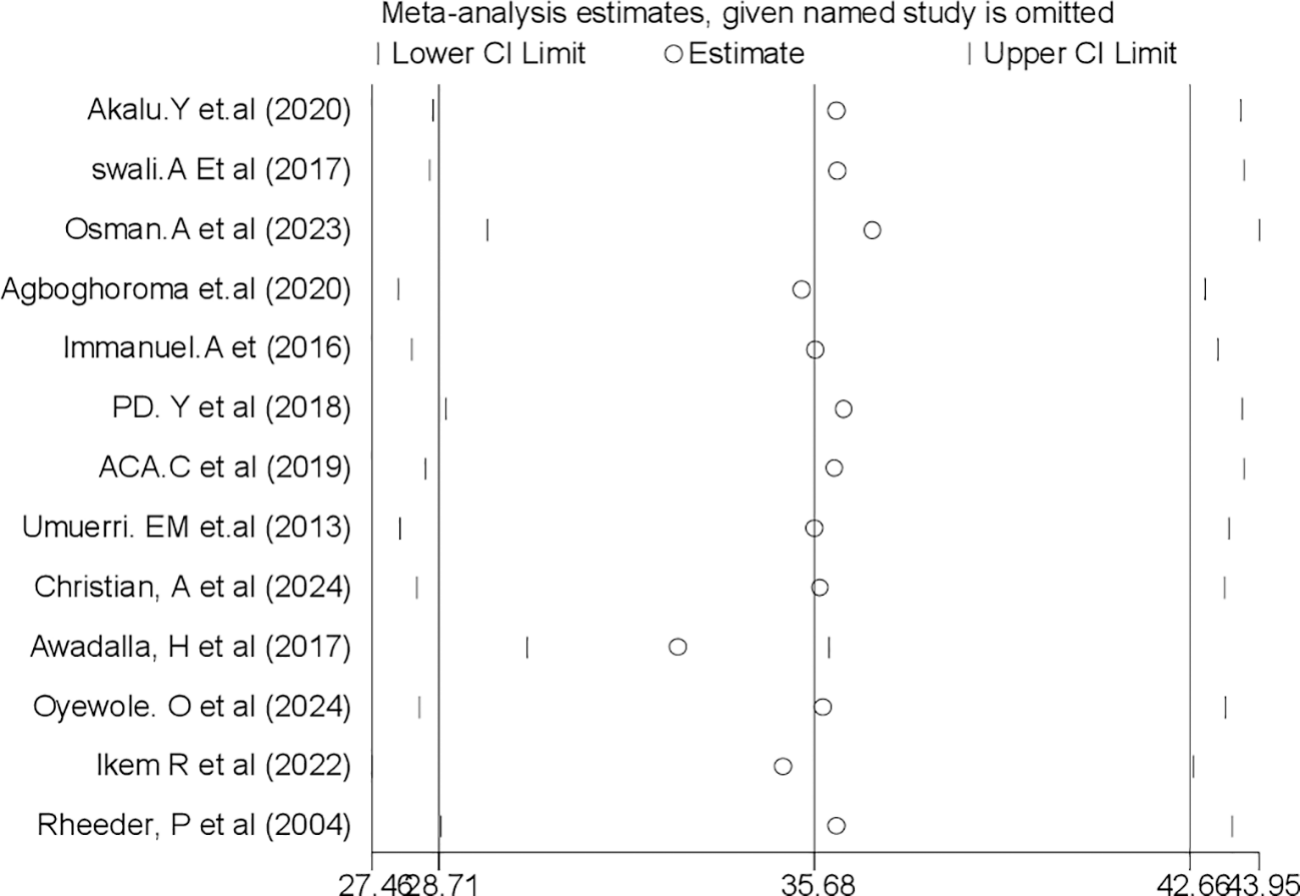
Sensitivity analysis for systematic review and meta-analysis of peripheral arterydisease among patients with T2DM in sub-Saharan Africa.

By excluding each study individually, a leave-out-one sensitivity analysis was used to assess the impact of each study on the pooled prevalence of PAD among patients with DM in sub-Saharan Africa. The findings revealed that the studies that were removed had no discernible impact on the pooled prevalence of PAD among patients with T2DM in sub-Saharan Africa (**Figure 5**).

#### Factors associated with the prevalence of PAD among patients with T2DM

To determine factors associated with PAD among patients with T2DM in sub-Saharan Africa, variables such as sex, age, diabetic foot ulcer, body mass index (BMI), duration of illness, hyperglycemia, occupation, history of leg pain, presence of comorbidities such as hypertension, smoking, hemoglobin A1C (HbA1c), low-density lipoprotein (LDL), patients on sulfonylurea–glibenclamide, intermittent claudication, and microalbuminuria were extracted from the relevant studies. Among these variables, four were identified as significant factors associated with PAD among patients with T2DM: age, BMI, duration of diabetes, and LDL (**Table 5**). However, in our study, there was no significant association between sex, smoking status, hypertension, diabetic foot ulcers, and history of leg pain with prevalence of PAD. To identify the association between age and PAD, three studies were included, all of which demonstrated a statistically significant association. Ultimately, our systematic review and meta-analysis revealed that individuals aged over 60 years had a 3.07-fold greater risk of developing PAD than individuals who were younger (OR = 3.07; 95% CI 1.68, 10.9). Moreover, the odds of developing PAD were 3.03 times greater in individuals with a BMI of obesity than in those with a normal BMI (OR = 3.03; 95% CI 1.7, 7.56). The odds of having a higher LDL were 3.6 times higher than those of individuals having a lower cholesterol level (OR = 3.6; 95% CI 1.5, 13.1). The length of time a patient was diagnosed with DM increased the likelihood of developing PAD by a factor of 2.44 (OR = 2.44; 95% CI 1.12, 5.13).

**Table 5:** Factors associated with peripheral artery disease among patients with T2DM in sub-Saharan Africa.

Determinants (Ref.No)	Number of studies	Sample size	OR(95% CI)	P-value	I2 (%)	Heterogeneity test(P-value)
Age [1-3]	3	747	3.072(1.68-10.9)	0.006	76.9%	0.013
Lowe density lipoprotein(LDL)[3-5]	3	786	3.6 (1.5-13.1)	0.047	74.9%	3.98
Body Mass Index (BMI) [2-4]	3	567	3.03(1.74-7.56)	<0.001	45.5%	1.83
Duration of DM [3, 6]	2	567	2.44(1.12-5.13)	0.024	28.4%	1.40

## Discussion

To determine the pooled burden of PAD among patients with T2DM in sub-Saharan Africa, we conducted a systematic review and meta-analysis. The reported prevalence of PAD among patients with T2DM in sub-Saharan Africa indicates a significant public health concern. This rate is notably higher than that observed in many high-income countries, where the prevalence of PAD among patients with diabetes typically ranges from 15% to 25%, which is greater than our finding. The elevated prevalence in sub-Saharan Africa may be attributed to a combination of factors, including genetic predispositions, lifestyle choices, and limited access to healthcare resources. The findings revealed that the pooled prevalence of PAD among patients with T2DM in sub-Saharan Africa was 35.68 (95% CI 28.7, 42.7). This finding was consistent with a study conducted in Ghana (24) and Gabon (32), which reported that the pooled prevalence of PAD among individuals with T2DM was 35.5% and 34.24%, respectively. The possible explanations could be the inclusion of studies within the continent of Africa, with most of the subcontinent having similar socio-demographic traits, poor self-care behaviors, low-income countries, culture, and poor accessibility of healthcare service-related factors.

Our aggregated prevalence systematic review and meta-analytic data concerning the burden of PAD among individuals with T2DM reveal figures that surpass those documented in pooled prevalence studies conducted in India (37), South Korea (38), and the United Kingdom (39), which indicated prevalence rates of 18%, 11.9%, and 6%, respectively. The reason for this disparity may be the fact that European and Asian countries have good screening programs, educated populations, easily accessible healthcare services, high-income levels, and better health career providers than countries in sub-Saharan African nations.

Conversely, the aggregated prevalence ascertained in our systematic review and meta-analysis was less than that indicated in a prior investigation concerning the prevalence of PAD among individuals diagnosed with T2DM in Nigeria (30), Sri Lanka (40), and Sudan (27), which was 42.5%, 65.5%, and 68.2%, respectively. A possible justification for this discrepancy is the difference in sample size, study, duration of follow-up, definitions of PAD, and study year. In general, the incidence and prevalence statistics pertaining to PAD exhibited considerable variability among the various studies in patients with T2DM. This variability may be partially ascribed to the disparate characteristics (study design, sample size, duration of follow-up, and definitions of PAD) of the studies that were incorporated.

According to our pooled findings, patients aged greater than 60 years had a 3.07-fold increased risk of developing PAD compared with their younger counterparts. This phenomenon may be attributed to the fact that as age increases, the prevalence of risk factors such as cardiovascular disease, chronic kidney disease, stroke morbidity, and mortality increases, all of which are associated with an escalation in PAD incidence. Generally, the older an individual is, the greater the probability of peripheral vascular compromise. This finding was consistent with studies conducted in Sudan, Ethiopia, India, Tanzania, and the United States (USA) (28, 31, 34, 41, 42). For instance, a study in the USA reported that the prevalence of PAD was 15.9% among individuals aged 60–69 years and 33.8% among those aged 70–82 years (41).

Our data also suggested that PAD was associated with the duration of DM with an odds of 2.44 times greater risk of developing; the result is consistent with a previous study done in Ghana, Tanzania, and Brazil (24, 28, 43). For instance, a study conducted in Tanzania revealed that a T2DM duration of more than 10 years was significantly associated with a 2.0-fold greater risk of developing PAD than participants with less than 10 years of DM duration (28). The plausible explanation could be that the most common level of arterial occlusion in PAD-associated diabetic feet is femoral–popliteal segment followed by tibia segment, which is a prominent risk factor for PAD.

A BMI exceeding a normal threshold level elevates the risk of developing PAD among patients with T2DM. Increased BMI is one of the contributing factors of PAD. Our findings indicate that individuals classified as obese are prone to developing PAD, which is 3.03 times greater. The results were aligned with studies conducted in Sudan, Nigeria, and Pakistan (30, 31, 44). It is presumed that obese patients have all the risk factors necessary to develop PAD. The other additional factors correlated with PAD include elevated LDL, with an OR of 3.6. These findings are supported by studies conducted in Brazil, Ethiopia, Nigeria, Sudan, Spain, Gabon, and Korea (27, 32, 43, 45–48). The possible justification is that elevated LDL affects fibrinolytic activity, increased circulating level of procoagulants such as tissue factor and factor IV, and decreased levels of anticoagulants like anti-thrombin-III and protein C, thus favoring a tendency toward coagulation, impaired fibrinolysis, endothelial wall dysfunction, and persistence thrombi, which are among the feasible mechanisms connecting PAD incidence (9, 29, 32).

### Clinical importance

Our findings indicate that the prevalence of PAD among patients with type 2 diabetes is significant, highlighting the need for greater emphasis on this issue. The research result also highlights specific risk factors related to PAD, which can inform targeted public health interventions and clinical strategies to mitigate the risk in T2DM populations. However, the intersection of these conditions necessitates a holistic approach to healthcare in the region.

### Strength of the study

The strength of our study lies in the fact that this was, to our knowledge, the first systematic review and meta-analysis to determine the overall pooled prevalence of PAD among individuals with T2DM in sub-Saharan Africa, and all the studies included were of good methodological quality. Furthermore, this systematic review and meta-analysis was performed and reported according to PRISMA guidelines.

### Limitations of the study

The presence of significant heterogeneity (more than 94%) in a small number of studies conducted on the study population may affect the generalizability of the findings. The included studies came from seven countries, further affecting the generalizability of the findings to the entire subcontinent. The included studies were cross-sectional for prevalence of factors; as a result, the outcome variable might be affected by other confounding variables, decreasing the power of the study and the causal conclusion between PAD and factors associated with a PAD. Studies with a small sample size for some included studies may be another limitation. The restriction of studies written in English, which limits the number of studies included in this meta-analysis, and the use of clinically healthy respondents may also lower the actual prevalence of PAD among individuals with T2DM. Additionally most studies from sub-Saharan Africa were hospital-based and might not be a reflection of the general population.

### Conclusion and recommendations

According to our systematic review and meta-analysis, the pooled burden of PAD was 35.7%, reflecting the significant impact of T2DM on vascular health. Exceeding 10 years duration of DM, advanced age, increased LDL level, and greater threshold level of BMI are all strong determinants of PAD among individuals with T2DM. Despite the alarming prevalence of PAD among patients with T2DM, it remains underdiagnosed; increased awareness, regular screening using the ABI for early detection, and a management strategy within the clinical setup for patients with diabetes are necessary. The need for integrated care approaches is mandatory. Future research should focus on longitudinal studies that explore the causal relationships between risk factors and the development of PAD in patients with T2DM.

## Data Availability

The original contributions presented in the study are included in the article/**Supplementary Material**. Further inquiries can be directed to the corresponding author.
