# Supplementary material for: Exploring the prevalence and risk factors of peripheral artery disease in patients with type 2 diabetes in sub-Saharan Africa: a systematic review and meta-analysis
Source: Front Clin Diabetes Healthc. 2025 Jul 21;6:1563984. doi: 10.3389/fcdhc.2025.1563984 (PMC12320937; doi:10.3389/fcdhc.2025.1563984)
Supplement: Supplementary file 1 [file SupplementaryFile1.docx]

**Supplementary File 1:** Search strategy for prevalence of PAD and associated factors among patients with T2DM in sub-Saharan Africa: A Systematic review and Meta-analysis.

| **Database** | **Example of searching strategy** | **Number of studies** |
| --- | --- | --- |
| (Science Direct, Medline, Cochrane Library , EMBASE, Scopus and AJOL) | ((("Peripheral artery disease *") OR ([lower extremity artery disease](https://www.sciencedirect.com/topics/medicine-and-dentistry/arteriosclerosis-obliterans))) OR (PAD) OR (arterial stiffness)) AND (("peoples))"[All Fields]) OR ("patients))*"[All Fields])) AND (((((sub-Saharan) OR ("Eastern Africa")) OR ("Western Africa")) OR ("Central Africa")) OR ("South Africa")). | 201 |
| Google Scholar | “Peripheral artery disease” OR “[lower extremity artery disease](https://www.sciencedirect.com/topics/medicine-and-dentistry/arteriosclerosis-obliterans)” OR “PAD” OR “arterial stiffness” AND "DM patients" OR "Type 2DM Individuals" AND sub-Saharan Africa". | 1798 |
